# Supplement Timing of Cranberry Extract Plays a Key Role in Promoting *Caenorhabditis elegans* Healthspan

**DOI:** 10.3390/nu6020911

**Published:** 2014-02-21

**Authors:** Sujay Guha, Ojas Natarajan, Cole G. Murbach, Jessica Dinh, Ethan C. Wilson, Min Cao, Sige Zou, Yuqing Dong

**Affiliations:** 1 Department of Biological Sciences, Clemson University, Clemson, SC 29634, USA; E-Mails: sguha@g.clemson.edu (S.G.); onatara@clemson.edu (O.N.); cmurbac@g.clemson.edu (C.G.M.); jdinh@g.clemson.edu (J.D.); pelamisplaturus@gmail.com (E.C.W.); mcao@clemson.edu (M.C.); 2 Institute for Engaged Aging, Clemson University, Clemson, SC 29634, USA; 3 Functional Genomics Unit, Translational Gerontology Branch, National Institute on Aging, Baltimore, MD 21224, USA; E-Mail: zous@grc.nia.nih.gov

**Keywords:** aging intervention, timing, nutraceuticals, cranberry polyphenols, healthspan, *Caenorhabditis elegans*

## Abstract

Consumption of nutraceuticals is a major and potent dietary intervention for delaying aging. As the timing of administration is critical for the efficacy of bioactive compounds in medicine, the effectiveness of nutraceuticals may also be dramatically affected by the timing of supplementation. Cranberry exact (CBE), rich in polyphenols, is consumed as a nutraceutical, and possesses anti-aging properties. Here, we examined the influence of timing on the beneficial effects of CBE supplementation in *C. elegans*. The prolongevity effect of CBE in different aged worms, young adults, middle-age adults, and aged adults, was determined. Early-start intervention with CBE prolonged the remaining lifespan of worms of different ages more robustly than late-start intervention. The effectiveness of CBE on stress responses and physiological behaviors in different aged worms was also investigated. The early-start intervention prominently promoted motility and resistance to heat shocks and *V. cholera* infection, especially in aged worms. Together, these findings suggest that the timing of CBE supplementation critically influences its beneficial effects on *C. elegans* lifespan and healthspan. It is of interest to further investigate whether the similar results would occur in humans.

## 1. Introduction

Numerous intrinsic and extrinsic factors affect the process of aging [1,2,3,4]*.* During the aging process, damages at the molecular, cellular, and organ level are accumulated, which in turn, results in a decline in a number of physiological, immunological, and cognitive functions. Therefore, it is critical to find ways to halt or delay aging processes. A growing body of evidence has suggested that diet has an enormous impact on health and lifespan in almost all organisms including humans [5,6,7]. Dietary intervention, with bioactive components from natural dietary sources, has shown immense promise in promoting healthy aging [1]. Many nutraceuticals, such as those from blueberry, curcumin, and Gingko, contain a variety of bioactive components and have garnered attention due to their potential health benefits [8,9,10,11].

Although dietary intervention is a non-genetic way to influence healthspan, bioactive food components may be functioning through genetic healthspan determinants, such as insulin signaling pathway, daf-16/FOXOs, and heat shock factors [1,2,4]. As aging is a complex process, many lifespan/healthspan-related genes may be involved in the developmental process and be expressed in a temporally and spatially regulated manner. Thus, the same functional diet may demonstrate very different efficacy when supplemented during animals’ developmental and mature stages; therefore, the timing of administration should be considered as a crucial determinant to ensure the effectiveness of functional diets.

The North American cranberry (*Vaccinium macrocarpon*) and its products, rich in polyphenols, have been used extensively as nutraceuticals owing largely to their anti-microbial, anti-mutagenic, anti-angiogenic, and anti-oxidant properties [12,13]. Recently, we reported that consumption of cranberry extract (CBE) rich in polyphenols dramatically extend lifespan in *C. elegans* and *D. melanogaster* [14,15]. Furthermore, we have shown that the prolongevity effect of CBE requires the insulin signaling pathway and the activities of DAF-16, and this effect is dose dependent in *C. elegans*. Healthspan assays indicate that CBE supplementation improves worms’ thermotolerance [14]. Although studies on CBE have demonstrated its benefits to healthy aging [16], information regarding the timing of CBE supplementation is still lacking.

Here, we examined the healthspan effect of CBE in different aged worms by altering the timing of CBE supplementation. Considering that *C. elegans* mean lifespan is around two weeks at 25 °C, we defined two-day old adult worms as young adults, six-day old adult worms as middle-age adults, and 10-day old adult worms as aged adults. Our data suggested that supplementation started from eggs hatching (defined as “early-start intervention”) prominently prolonged the remaining lifespan in all three different aged populations (young adults, middle-age adults, and aged adults) relative to supplementation started from L4 stage (defined as “late-start intervention”). We distinguished that the early developmental stage played a more critical role in CBE-mediated prolongevity relative to CBE long-term treatment. We further examined worms’ responses to heat shock and *V. cholerae* infection and their physiological locomotion using similar timing strategies. Our results similarly indicated that the early-start intervention leads to more prominent benefits to *C. elegans* overall health status as compared to the late-start intervention. Briefly, the CBE early-start intervention significantly improved their motility and heat shock tolerance, especially in aged worms. The early-start intervention, not the late-start intervention, has the significant efficacy in preventing *V. cholerae* infection in *C. elegans*. Taken together, our findings reveal that CBE supplementation starting at the early developmental stages is critical to optimizing its benefits in *C. elegans* healthspan.

## 2. Materials and Methods

### 2.1. CBE Preparation

The CBE, rich in polyphenols, kindly provided by Dan Souza (Decas Botanical Synergies, Carver, MA, USA), was described in a previously published study [14]. To prepare cranberry supplemented food, the CBE in powder was first dissolved in sterile distilled water. A fresh 10% (w/v) stock solution was prepared one day before the assay and then the appropriate dilutions were overlaid onto nematode growth medium (NGM) plates.

### 2.2. *C. elegans* Maintenance

Wild type N2 worms were obtained from the *Caenorhabditis* Genetics Center (CGC), University of Minnesota, Minneapolis, MN, USA and maintained at 20 °C on NGM seeded with *E. coli* OP50 feeding strain. A volume of 100 µL of OP50 (overnight culture) was dropped on the center of 60-mm NGM plates, which were allowed to dry overnight before worms were transferred.

### 2.3. Lifespan and Remaining Lifespan Assay

All assays of lifespan and remaining lifespan were carried out at 25 °C. Synchronized populations were obtained by allowing 10–15 hermaphrodites lay eggs overnight at 16 °C onto *E. coli* OP50 seeded regular NGM plates or treatment plates (NGM plates containing 2 mg/mL CBE), and then the parents were removed the next day. The eggs were allowed to hatch and develop until reaching L4 stage.

To carry out early-start intervention with CBE, synchronized L4 stage worms from treatment plates were transferred onto a modified treatment plate (treatment plate containing 50 μg/mL FUDR to prevent the growth of progeny) seeded with *E. coli* OP50 and then continue to grow for 2 days, 6 days, and 10 days, respectively. For the late-start intervention with CBE, synchronized L4 stage worms from regular NGM plates were transferred onto a modified treatment plate seeded with *E. coli* OP50 and continue to grow for 2 days, 6 days, and 10 days, respectively.

For remaining lifespan assays, 30 worms from each time point after either early-start intervention or late-start intervention, were transferred to the FUDR-containing NGM plates and remaining lifespan assays were carried out until all worms died. Worms of the same ages from regular NGM plates without CBE intervention were transferred onto a FUDR-containing NGM plate as controls. The dead worms were counted starting the day after worms finished CBE intervention and exploding, protruding, bagging or contaminated worms were censored if applicable. We defined the day when the worms finished early-start intervention and late-start intervention as day 0 of the remaining lifespan. *E. coli* OP50 used for remaining lifespan assays was 10-fold concentrated from overnight culture by centrifugation.

For early-start intervention mediated lifespan assays, 30 L4 stage worms from treatment plates were transferred onto a modified treatment plate seeded with *E. coli* OP50 to begin the lifespan assay. Leftover worms after 2 days, 6 days, and 10 days, were, respectively, transferred to the FUDR-containing NGM plates without CBE, and the lifespan assays continued until all worms died. Synchronized L4 worms from regular NGM plates were transferred onto the FUDR-containing NGM plates as controls. The dead worms were counted starting the next day after L4 worms were transferred and exploding, protruding, bagging or contaminated worms were censored if applicable. We defined the day when we transferred L4 worms as day 0 of the lifespan. *E. coli* OP50 used for lifespan assay was 10-fold concentrated from overnight culture by centrifugation.

All the assays were carried out in triplicates and a minimum of two independent trials were performed for all conditions. All statistical analyses were carried out using SPSS software (IBM SPSS Statistics, Armonk, NY, USA). Kaplan-Meier lifespan analysis was carried out, and *p* values were calculated using the log-rank test, *p* < 0.05 was accepted as statistically significant. The significant different groups remained statistically significant at *p* < 0.05 after Bonferroni corrections for multiple comparisons (data not shown).

### 2.4. Heat Shock Assay

Synchronized worms, after either early-start intervention or late-start intervention, were harvested and transferred to regular NGM plates. Worms on regular NGM plates without CBE intervention served as controls. The plates were incubated at 37 °C for 1.5 h and then transferred back to 25 °C. The survival of the worms was monitored daily until all worms died. Each assay was carried out in three independent trials, and the mean survival time (lifespan) was analyzed using SPSS software (IBM SPSS Statistics). Kaplan-Meier lifespan analysis was carried out and *p*-values were calculated using the log-rank test, *p* < 0.05 was accepted as statistically significant. On the basis of lifespan assays, the percentage increases of mean survival time were calculated and pooled.

### 2.5. Bacterial Killing Assay

*V. cholerae* killing assays were carried out by performing the remaining lifespan assay on FUDR-containing NGM plates seeded with *V. cholerae* wild type C6706 (O1 Biotype El Tor strain), rather than OP50. *V. cholerae* used for killing assay was 10-fold concentrated from overnight culture by centrifugation.

### 2.6. Motility Assays

Ten CBE-treated (early intervention and later intervention) or non-treated day 2, day 6, and day 10 adult nematodes were placed onto individual NGM plates without OP50. The body bends per minute were counted. This assay was carried out in three independent trials. The data were pooled and analyzed using Student’s *t* test, *p* < 0.05 was accepted as statistically significant.

## 3. Results and Discussion

### 3.1. Early-Start Intervention with CBE Shows More Potency in Increasing the Remaining Lifespan of Elderly Worms Relative to the Late-Start Intervention

**Figure 1:**
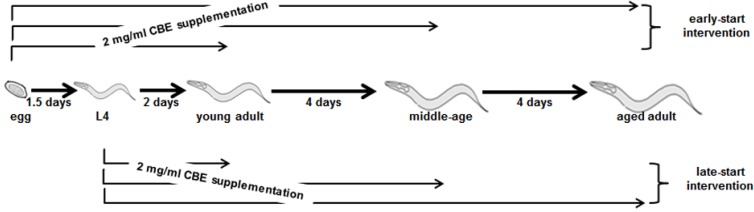
A scheme of the early-start intervention and late-start intervention.

To determine the best timing of CBE supplementation, we compared the prolongevity efficacy of early-start intervention and late-start intervention in different aged worms. The early-start intervention initialized the CBE treatment prior to eggs hatching, and continued until reaching young adult, middle-age adult, and aged adult. The late-start intervention began the CBE treatment from L4 stage and continued to young adult, middle-age adult, and aged adult. We summarized the intervention methods in Figure 1. Thereafter, we measured the remaining lifespan of young adult, middle-age adult, and aged adult, respectively. For the early-start intervention to young adult worms, the remaining lifespan was prolonged from 10.1 days to 11.2 days, which increased 11.1% (Table 1), while the late-start intervention prolonged the remaining lifespan of young adults from 10.1 days to 10.9 days, an increase of only 8.2% (Table 1). A 32.7% increase of remaining lifespan was observed, in the early-start intervention to middle-age adults, while the late-start intervention increased 21.2% (Table 1). Impressively, the early-start intervention to aged adults resulted in an 80.8% increase in remaining lifespan, and the late-start intervention led to a 69.7% increase (Table 1). Our data indicated that although the late-start intervention could extend the remaining lifespan in different aged worms, the early-start intervention overall showed greater efficacy. Most notably, our data clearly showed that CBE supplementation lasting until the worms were aged dramatically elevated the remaining lifespan of the aged population, especially the early-start intervention (Table 1). These findings suggest that in order to fully exert CBE’s prolongevity effects, CBE supplementation should start in the early developmental stage and be continued in a long-term manner.

**Table 1:** The remaining lifespan of different aged worms at 25 °C. Lifespan and standard error are shown in days.

Treatment	Mean ± SE	Median	# of Worms	Increase%	*p*-Value
Young adult control	10.1 ± 0.3	10.0	118	-	-
EI to young adult	11.2 ± 0.2	11.0	116	11.1%	0.001
LI to young adult	10.9 ± 0.2	11.0	111	8.2%	0.006
Middle-age control	7.0 ± 0.2	7.0	115	-	-
EI to middle-age	9.3 ± 0.3	9.0	117	32.7%	0.001
LI to middle-age	8.5 ± 0.7	9.0	103	21.2%	0.001
Aged adult control	3.1 ± 0.2	3.0	89	-	-
EI to aged adult	5.6 ± 0.2	6.0	87	80.8%	0.001
LI to aged adult	5.2 ± 0.3	5.0	84	69.7%	0.001

The remaining lifespan experiments were repeated at least three times with similar results, and the data for representative experiments are shown. The lifespan data were analyzed using the log-rank test and *p*-values for each individual experiment are shown. EI represents the early-start intervention. LI represents the late-start intervention. # indicates number.

### 3.2. Early Developmental Stages of *C. elegans* Play More Prominent Role in CBE-Mediated Lifespan Extension as Compared to CBE Long-Term Supplementation

**Figure 2:**
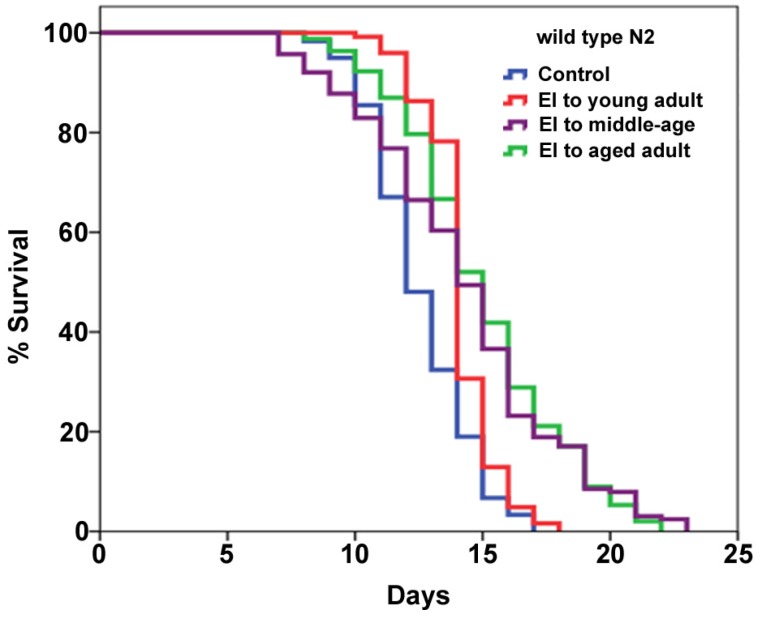
Longer time supplementation with CBE results in more prolonged lifespan extension in *C. elegans*. Wild type N2 worms were supplemented with 2 mg/mL of CBE through the early-start intervention. Each lifespan experiment was repeated at least three independent times with similar results. Quantitative data and statistical analyses for the representative experiments are included in Table 2. EI represents the early-start intervention.

As both of the early developmental stage and long-term treatment contributed to the CBE-mediated prolongevity, we next wanted to assess and compare their prolongevity potency. To this end, we carried out a regular lifespan assay to measure the effectiveness of each early-start intervention method (Table 2, Figure 2). Compared to untreated worms, *C. elegans* treated with CBE from egg until young adult, increased the mean lifespan of worms from 12.5 days to 14.1 days, resulting in 12.8% increase. The early-start intervention to middle-age prolongs the mean lifespan to 14.3 days (14.4% increase), and the early-start intervention to aged adult prolongs the mean lifespan up to 15.0 days (20.0% increase). Considering that the larval stage from egg to L4 only takes ~1.5 days (36~40 h) at 25 °C, the early-start intervention to young, middle-age, and aged adults takes around 3.5, 7.5, and 11.5 days, respectively. Compared to the 12.8% increase (intervention to young adults), the intervention to aged adults took eight more days but only contributed an additional 7.2% increase (20% increase in total). Hence, our results imply that the early developmental stage plays more prominent role in CBE-mediated lifespan extension as compared to CBE long-term supplementation.

**Table 2:** The lifespan of worms at 25 °C with various early-start interventions. Lifespan and standard error are shown in days.

Treatment	Mean ± SE	Median	# of Worms	*p*-Value
N2 control	12.5 ± 1.0	13.0	179	
EI to young adult	14.1 ± 1.2	14.0	141	0.001
EI to middle-age adult	14.3 ± 0.9	14.0	164	0.001
EI to aged adult	15.0 ± 1.2	15.0	146	0.001

Results presented in Figure 2. The lifespan experiments were repeated at least three times with similar results, and the data for representative experiments are shown. The lifespan data were analyzed using the log-rank test and *p*-values for each individual experiment are shown. EI represents the early-start intervention. # indicates number.

### 3.3. Influence of CBE Early-Start Intervention and Late-Start Intervention on Worms’ Heat Shock Resistance and Motility

**Figure 3:**
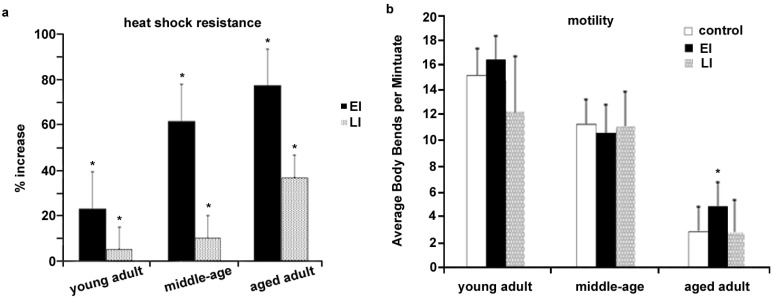
Outcomes of the early-start intervention and the late-start intervention on *C. elegans* heat shock resistance and motility. (**a**) N2 worms after either the early-start intervention or the late started intervention exhibited increased survival after 1.5 h at 37 °C when compared to control worms; (**b**) only worms after the early-start intervention showed an elevated motility rate in aged population, as compared to control worms. At least triplicate samples were examined for each stress assay. “% increase” indicates the average increase among the multi-replicates and error bars represent the standard deviation. *p* value was calculated using Studentʼs t-test. * *p* < 0.05 when compared to control. Each of the stress assays was repeated at least three independent times with similar results. EI represents the early-start intervention; LI represents the late-start intervention.

Considering the correlation between prolongevity and better health conditions, we examined *C. elegans* heat shock resistance after either early-start intervention or late-start intervention. Heat shock resistance has been coupled with the benefit of CBE-mediated prolongevity [14]. Our results indicate that both early-start and late-start interventions resulted in statistically significant resistance to heat shock in each age group, as compared to the controls without CBE treatment (Figure 3a). In addition, the early-start intervention shows much stronger potency relative to the late-start intervention (Figure 3a), suggesting that the larval stages (early developmental stage) are important for developing CBE-mediated heat shock resistance. We cannot rule out the possibility that the longer time of intervention may also be critical to improving *C. elegans* heat shock resistance, as for both interventions there was a positive correlation between length of intervention and increased resistance. However, again, considering that the larval stage from egg to L4 only takes ~1.5 days (36–40 h) at 25 °C, the early-start intervention to middle-age adult will take ~7.5 days, while the late-start intervention to middle-age adult takes six days. This 1.5 days of difference led to a resistance increase from 9.3% (late-start) to 61.3% (early-start) (Figure 3a). This comparison clearly implied that the early developmental stage is important for CBE-mediated heat shock resistance. Similarly, the early-start intervention to aged adult will take ~11.5 days. As compared to the late-start intervention to the aged adult (10 days), the 1.5 days of difference led to the resistance increase from 33.8% (late-start) to 76.5% (early-start), which further confirmed our conclusion that the larval stage is important for establishing CBE-mediated heat shock resistance.

Motility is one of the key physiological indexes to assess animals’ degree of aging. Given the evidence that both of the early-start and late-start interventions to aged worms can dramatically increase the remaining lifespan (Table 1), we postulated that the early-start and the late-start interventions to aged adults would improve locomotion in aged populations. Surprisingly, only the early-start intervention significantly improved the motility of aged worms. The late-start intervention did not benefit aged worms’ motility (Figure 3b).

Taken together, our findings from the heat shock assay and motility assay imply that aging intervention, if started at the early stage of development, may significantly influence healthy aging in aged populations. This is reasonable since the developmental period before adulthood is extremely important in determining an animal’s long-term health [17].

### 3.4. Protective Effects of CBE Supplementation on *Vibrio cholerae* Killing in *C. elegans*

Bacterial infection is a leading cause of death among the elderly [18]. We found that CBE supplementation could protect worms from *V. cholerae* infection (unpublished data) and this protection is not due to the antibacterial effects of CBE (data not shown). Here, we determined how CBE may lead to the best protective effects against *V. cholerae*. We performed the *V. cholerae* killing assays in different aged worms. All three of the early-start intervention groups were significantly protected against *V. cholerae* killing (Table 3). Conversely, the late-start intervention did not show the significant protection in the young, middle-age, or aged worms (Table 3). It is known that the developmental stage before adulthood is an essential period for an animal to develop its immune system [19]. *C. elegans* feeds on bacteria and has evolved and maintained an innate immune system to defend against pathogenic attack [20]. Therefore, the difference of the early-start intervention and the late-start intervention shown on *V. cholerae* resistance may indicate that CBE helps *C. elegans* build up a robust innate immunity in the larval stage. Undoubtedly, to support this hypothesis, more worm pathogens need to be tested in further experiments.

**Table 3:** The survival time of different aged worms after exposed to *V. cholera* at 25 °C. Lifespan and standard error are shown in days.

Treatment	Mean ± SE	Median	# of Worms	Increase%	*p*-Value
Young adult control	7.5 ± 1.1	7.0	213	-	-
EI to young adult	8.3 ± 1.9	8.0	216	11.5%	0.001
LI to young adult	7.7 ± 1.2	8.0	163	-	0.890
Middle-age control	5.5 ± 1.2	5.0	213	-	-
EI to middle-age	6.8 ± 1.2	7.0	197	23.3%	0.001
LI to middle-age	5.5 ± 0.7	6.0	173	-	0.987
Aged adult control	4.5 ± 1.2	4.0	204	-	-
EI to aged adult	5.6 ± 1.1	6.0	169	25.3%	0.001
LI to aged adult	4.6 ± 0.8	4.0	181	-	0.197

The *V. cholera* killing assays were repeated at least three times with similar results, and the data for representative experiments are shown. The lifespan data were analyzed using the log-rank test and *p*-values for each individual experiment are shown. EI represents the early-start intervention. LI represents the late-start intervention. # indicates number.

## 4. Conclusions

We dissected the timing of CBE application in the present studies and our findings revealed the importance of the early developmental stage before adulthood in aging intervention with CBE. The early-start intervention with CBE significantly and dramatically promoted *C. elegans* healthspan, especially in aged populations. On basis of our experimental evidence, we are the first to suggest how to implement CBE to optimize its prolongevity effects in a multicellular organism. That is, to promote healthspan, CBE supplementation/intervention should start at the early stage of development and, it is better if the intervention is applied in a long-term manner. Considering that CBE-mediated prolongevity and stress responses require evolutionarily conserved mechanisms among diverse species ranging from *C. elegans* to mammals [14,21,22], our findings have imperative implications for the application of CBE in improving healthspan in higher order organisms, including humans. Noticeably, however, if the similar results would occur in humans still remains unknown.
